# Oscillatory Corticospinal Activity during Static Contraction of Ankle Muscles Is Reduced in Healthy Old versus Young Adults

**DOI:** 10.1155/2018/3432649

**Published:** 2018-04-26

**Authors:** Meaghan Elizabeth Spedden, Jens Bo Nielsen, Svend Sparre Geertsen

**Affiliations:** ^1^Department of Nutrition, Exercise and Sports, University of Copenhagen, Copenhagen, Denmark; ^2^Department of Neuroscience, University of Copenhagen, Copenhagen, Denmark; ^3^Elsass Institute, Charlottenlund, Denmark

## Abstract

Aging is accompanied by impaired motor function, but age-related changes in neural networks responsible for generating movement are not well understood. We aimed to investigate the functional oscillatory coupling between activity in the sensorimotor cortex and ankle muscles during static contraction. Fifteen young (20–26 yr) and fifteen older (65–73 yr) subjects were instructed to match a target force by performing static ankle dorsi- or plantar flexion, while electroencephalographic (EEG) activity was recorded from the cortex and electromyographic (EMG) activity was recorded from dorsi- (proximal and distal anterior tibia) and plantar (soleus and medial gastrocnemius) flexor muscles. EEG-EMG and EMG-EMG beta band (15–35 Hz) coherence was analyzed as an index of corticospinal activity. Our results demonstrated that beta cortico-, intra-, and intermuscular coherence was reduced in old versus young subjects during static contractions. Old subjects demonstrated significantly greater error than young subjects while matching target forces, but force precision was not related to beta coherence. We interpret this as an age-related decrease in effective oscillatory corticospinal activity during steady-state motor output. Additionally, our data indicate a potential effect of alpha coherence and tremor on performance. These results may be instrumental in developing new interventions to strengthen sensorimotor control in elderly subjects.

## 1. Introduction

Aging is accompanied by increased incidence of gait and balance problems, greater movement variability, reduced force steadiness, and worsened coordination [1–3], but age-related changes in neural control of motor function have yet to be fully elucidated. Previous studies have primarily focused on characterizing structural mechanisms, whereas knowledge regarding functional changes in the neural networks involved in generating movements is limited. A better understanding of how activity patterns in descending tracts controlling motor function are modified with age is of particular importance, as these tracts directly mediate motor signals from the brain to the spinal cord.

The corticospinal tract is a major descending pathway governing voluntary movement in humans and appears to be vulnerable to age-related deterioration. Diffusion tensor imaging has revealed declining fractional anisotropy (FA) in the posterior limb of the internal capsule, indicating degeneration in white matter microstructure [4, 5]. The functional significance of these declines is supported by associations between internal capsule FA and motor performance [6]. Input-output characteristics of the corticospinal system also appear to be modulated with age. Studies using transcranial magnetic stimulation indicate that the amplitude of motor-evoked responses is lower in old than in young adults [7–9], which suggests that aging may entail reduced excitability in the corticospinal pathway.

Another functional marker of corticospinal integrity is corticomuscular coherence, which quantifies the degree of task-related oscillatory coupling between the sensorimotor cortex and contralateral muscle. In healthy young adults, coherent oscillations are present between the cortex and muscle at beta frequencies (15–35 Hz) during isometric contractions and are mediated by the corticospinal tract [10–16]. Coherent beta oscillations may also be seen between different pools of motor neurons in synergists and within the same muscle [15, 17–19]. This intra- and intermuscular coherence shares several similarities with corticomuscular coherence and is generally assumed to be generated by similar mechanisms, and both inter- and intramuscular coherence are therefore often used as an indirect measure of the corticospinal activity [18, 19]. Coherent oscillations are also present in the alpha band (5–15 Hz) between pairs of motor units [17] but are only rarely seen between the cortex and the muscles [20, 21]. The origin of alpha coherence is debated, but some evidence suggests a central origin [21] and a relation to physiological tremor [22].

Previous investigations of age-related changes in beta band coherence have produced equivocal results. Studies have demonstrated reduced [23], increased [24], and unchanged [25] amplitudes of corticomuscular coherence in old versus young adults, whereas studies examining inter- or intramuscular coherence have shown both unchanged [26, 27] and increased [27, 28] magnitudes. However, the use of different motor tasks and muscle groups may render direct comparisons across studies inappropriate or, in the least, less straightforward. Studies exploring age-related changes in coherence have primarily focused on hand and arm muscles [23–25, 28]. Less is known about the cortical control of ankle muscles, which is of interest due to their important role in postural control and gait. For example, during gait, ankle dorsiflexors lift the toes in order to clear the ground in the swing phase, and plantar flexors play a crucial role in forward propulsion during the late stance phase [29]. To date, age-related changes in intermuscular coherence in ankle muscles during a simple phasic task [26] and during uni- and bipedal stance [27] have been explored, but investigation of the direct corticomuscular coupling is lacking.

We aimed to investigate cortico-, intra-, and intermuscular coherence in ankle muscles during static dorsi- and plantar flexion in old versus young adults. It was hypothesized that older adults would display less beta coherence than young adults, indicative of reduced oscillatory corticospinal activity. Additionally, we wanted to explore if beta coherence estimates were positively associated with task performance.

## 2. Methods

### 2.1. Subjects

Fifteen young (mean: 22.1 ± 1.7, range 20–26 yr; 7 men) and fifteen older (mean: 68.3 ± 2.7, range 65–73 yr; 7 men) participants were recruited (see Table 1). The sample size was chosen based on previous studies comparing coherence between groups [24, 25]. Subjects were free from neurological disorders and afflictions impairing leg motor function. No subjects were taking medication expected to affect neuromuscular function. There was no indication of cognitive impairment in any subjects as determined by the Mini-Mental State Examination (MMSE; all scores ≥ 26 out of 30) [30]. According to the Waterloo Footedness Questionnaire [31], 21 subjects were right-footed, 2 were left-footed, and 7 were equally right- and left-footed.

Prior to participation in the study, subjects provided written, informed consent. All procedures were approved by the ethics committee for the Capital Region of Denmark (approval number H-16021214), and experiments were conducted in accordance with the Helsinki declaration.

### 2.2. Protocol

Subjects were seated in a rigid chair with the left foot firmly fastened to a force pedal containing a strain gauge that measured force exerted on the pedal. The leg was positioned with ~90° flexion at the hip joint, ~115° flexion at the knee joint, and ~120° dorsiflexion at the ankle joint.

First, subjects performed three maximal isometric dorsiflexion contractions (MVC), separated by 30 s rest, as a measure of maximal voluntary dorsiflexion strength. Online visual feedback of force production was projected onto the wall in front of subjects as a moving yellow trace on a black background. The projector (TDP-T 255; Toshiba) was attached to the ceiling 148 cm from the wall, resulting in a screen size of 125 cm by 167 cm. During MVC, verbal encouragement was provided to ensure maximal efforts. MVC was determined as peak force production across the three trials.

**Figure 1:**
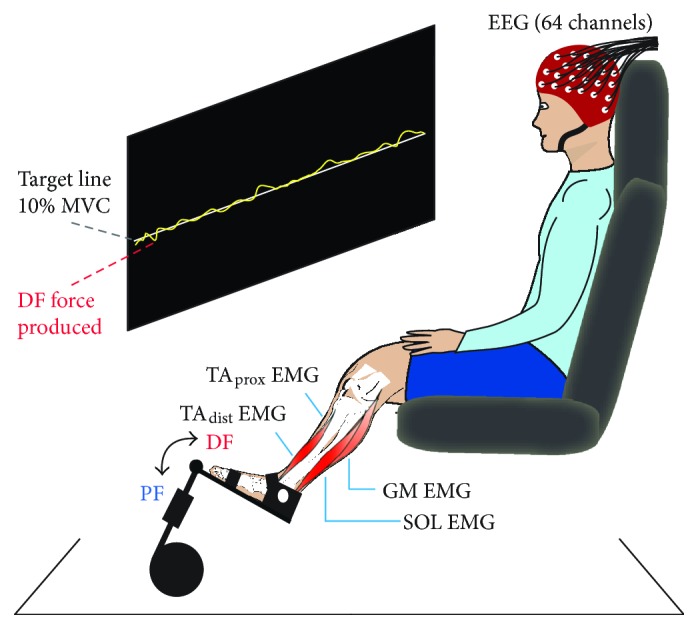
Experimental setup and static contractions. Subjects sat in a chair with their left foot fastened to a force pedal and maintained a force level of 10% of their maximal voluntary contraction (MVC) for 2 min, first for dorsiflexion (DF) and subsequently for plantar flexion (PF). The target force level was projected onto the wall as a horizontal line that subjects were asked to follow as precisely as possible with a yellow force trace showing online force production. During contractions, electroencephalographic (EEG) and electromyographic (EMG) signals from the proximal and distal ends of the anterior tibial muscle (TA_prox_ and TA_dist_), soleus (SOL), and medial gastrocnemius (GM) were recorded.

Subsequently, subjects performed a static contraction, where they were asked to maintain a force level of 10% MVC during isometric dorsiflexion for two minutes. The target force level was displayed on the wall as a horizontal line, and subjects were instructed to follow this line as precisely as possible with the moving yellow trace depicting real-time force production (Figure 1).

Following a short rest, subjects repeated the MVC procedure and static contraction for plantar flexion. During periods where data was recorded, subjects were asked to relax face and neck muscles to minimize artifacts in electroencephalographic (EEG) signals.

### 2.3. Electrophysiological Recordings

EEG and electromyographic (EMG) activity were recorded using active surface electrodes (BioSemi, The Netherlands) and ActiView acquisition software (version 6.05). EEG was measured using 64 pin electrodes plugged into a headcap (BioSemi) that ensured positioning in accordance with the 10/20 layout system. EMG was recorded from four pairs of electrodes placed on left lower leg muscles (interelectrode distance ~ 1.5 cm) after preparation of the skin with shaving, abrasion, and cleaning with alcohol. Pairs of EMG electrodes were positioned over the proximal (TA_prox_) and distal (TA_dist_) ends of the anterior tibial muscle (mean distance between electrode pairs ~ 12 cm) and over the center of the medial gastrocnemius (MG) and soleus (SOL) muscles. As per the BioSemi system design, signals were recorded in reference to a common mode sense active electrode, while a driven right leg electrode was used to cancel signals measured at the common mode sense electrode. Analog signals were sampled at 2048 Hz, and offset was contained to ±25 *μ*V.

### 2.4. Data Analysis and Statistics

All data analysis was performed in MATLAB (Version R2016b, MathWorks, MA, USA). Electrophysiological data was preprocessed using the EEGLAB toolbox (v14.0.0). Initially, EEG and EMG signals were downsampled to 256 Hz. EEG signals were then rereferenced to an average reference excluding noisy and high impedance channels and band pass filtered between 0.5 to 120 Hz. Independent component analysis was performed in EEGLAB [32] and was used to remove EEG signal components displaying unequivocal spatial and temporal characteristics of eye blinks and/or face and neck muscle activity [33]. EMG signals were band pass filtered between 5 and 120 Hz then full-wave rectified to emphasize information about timing of motor unit action potentials in the signals [34] and to improve coherence detection [35].

In order to examine the coupling between EEG-EMG and EMG-EMG signals in the frequency domain, coherence estimates were constructed [36]. Coherence describes the linear association between two signals at each frequency of interest and is a measure of phase consistency between signals. Coherence estimates are defined over the range [0, 1] where 0 indicates no association between signals and 1 indicates a strong association. To calculate coherence, auto- and cross-spectra were constructed by dividing signals into nonoverlapping segments, after which discrete Fourier transforms were performed on each segment and averaged. Coherence was then determined as the squared modulus of the cross-spectrum for the two signals *f*_*xy*_(*λ*) normalized by the product of the two autospectra, *f*_*xx*_(*λ*) and *f*_*yy*_(*λ*):

(1) $$\begin{array}{c} {\left|Rxy\left(\lambda \right)\right|}^{2}=\frac{{\left|f_{xy}\left(\lambda \right)\right|}^{2}}{f_{xx}\left(\lambda \right)f_{yy}\left(\lambda \right)}. \end{array}$$

EEG-EMG coherence thus quantifies the strength and frequency range of the coupling between cortical and muscular activity, whereas EMG-EMG coherence quantifies common rhythmic drive to two muscles or motor neuron pools. The statistical significance of individual coherence estimates was assessed according to an upper 95% confidence limit determined as

(2) $$\begin{array}{c} 1-0.05^{\frac{1}{\left(L-1\right)}}, \end{array}$$

where *L* indicates the number of data segments used for the analysis [36].

For data obtained during static dorsiflexion, coherence was calculated between EEG and TA_prox_; EEG and TA_dist_; and between TA_prox_ and TA_dist_. For static plantar flexion, coherence analysis was likewise performed for EEG and SOL; EEG and MG; and between SOL and MG. For all analyses, 120 seconds of data and a frequency resolution of 1 Hz were utilized.

**Figure 2:**
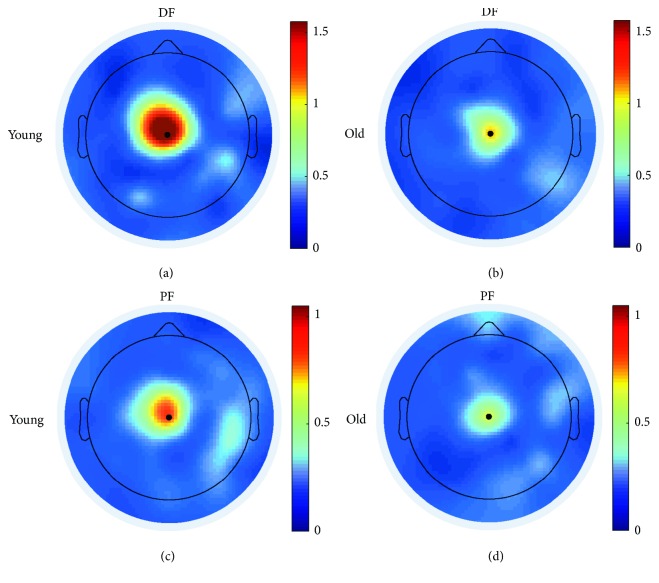
Coherence head plots. Spatial localization of summed beta band (15–35 Hz) corticomuscular coherence for single young (a, c) and old (b, d) subjects during static dorsiflexion (a, b; EEG-TA_prox_ coherence) and plantar flexion (c, d; EEG-SOL coherence).

**Figure 3:**
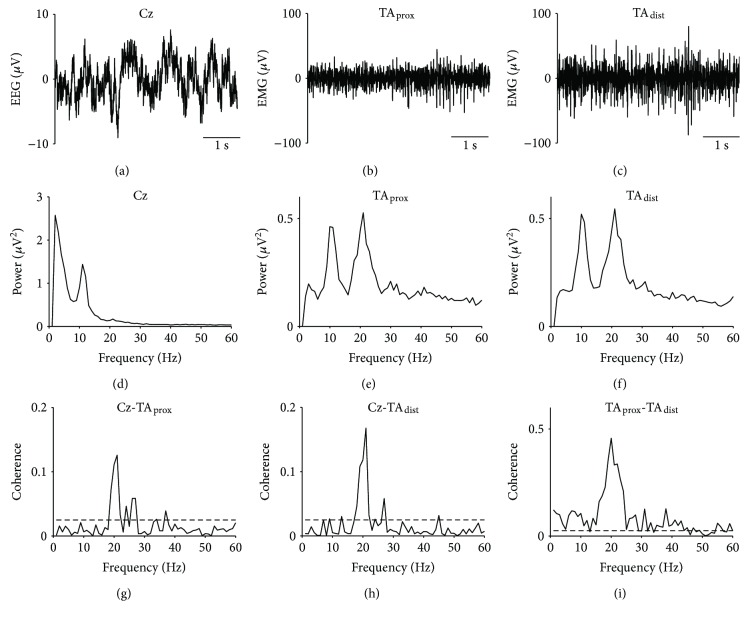
Raw EEG and EMG traces, autospectra, and coherence results from a single young subject. EEG from Cz (a), EMG from proximal (b), and distal (c) ends of the anterior tibial muscle (TA_prox_ and TA_dist_, resp.); autospectra for Cz (d), TA_prox_ (e), TA_dist_ (f); and coherence for Cz-TA_prox_ (g), Cz-TA_dist_ (h), and TA_prox_-TA_dist_ (i) during static dorsiflexion. Dashed lines on coherence plots indicate upper 95% confidence interval limits.

Initially, EEG-EMG coherence was calculated for all EEG electrodes. Scalp plots indicated that beta band (15–35 Hz) coherence was localized at the vertex (Cz), assumedly corresponding to the sensorimotor cortex (Figure 2). Accordingly, further analysis was performed using coherence at the Cz electrode (see example in Figure 3).

All EMG-EMG coherence data was visually inspected for signs of crosstalk, that is, high coherence across a wide range of frequencies and close to zero lag synchronization in the time domain as evidenced in cumulant density plots [37]. No data was observed displaying these characteristics, so all data was included in pooled analyses.

To undertake a population analysis, coherence for each age group was pooled, resulting in single group estimates at each frequency of interest. Group differences were investigated using the *χ*^2^ extended difference of coherence test [38], a nonparametric test that provides the amount of pooled coherence difference between groups at each frequency in relation to an upper 95% confidence interval limit.

Coherence was also quantified as the sum (i.e., area) of alpha (5–15 Hz), total beta (15–35), low (15–25 Hz), and high (25–35 Hz) beta coherence. These values were transformed logarithmically to symmetrize distributions for statistical analyses. Additionally, we registered the frequency at which peak beta coherence occurred as well as the sum of EMG power in alpha and beta bands.

Performance during static contractions was quantified as the root mean square (RMS) error, that is, the RMS deviation of the raw force signal from the target force level.

One-way ANOVA was used to evaluate effects of age group on dependent variables, that is, logarithmic coherence area estimates, frequency for peak beta coherence, summed EMG power, RMS error, and subject characteristics (MMSE score, height, weight, and body mass index (BMI)). Model assumptions were checked by visual inspection of residual and normal probability plots. ANOVA was also utilized to explore if gender and/or laterality (footedness) affected the area of beta coherence.

Associations between task performance and coherence estimates, as well as performance and power were assessed by means of Pearson correlations. The significance level used to reject the null hypothesis was *α* = 0.05, but tendencies were also noted for *p* < 0.1. Values are presented as mean ± standard deviation where applicable.

## 3. Results

Subject characteristics are summarized in Table 1. Young and old subjects were comparable in height (*p* = 0.349), body mass (*p* = 0.133), and MMSE score (*p* = 0.726), whereas older subjects had significantly greater BMI (*p* = 0.018).

EEG, EMG, autospectra, and coherence results from a single young subject during static dorsiflexion are shown in Figure 3. For this subject, sizeable coherence peaks were observed in the beta band (~20 Hz) for both cortico- and intramuscular coherence.

### 3.1. EMG Power

**Figure 4:**
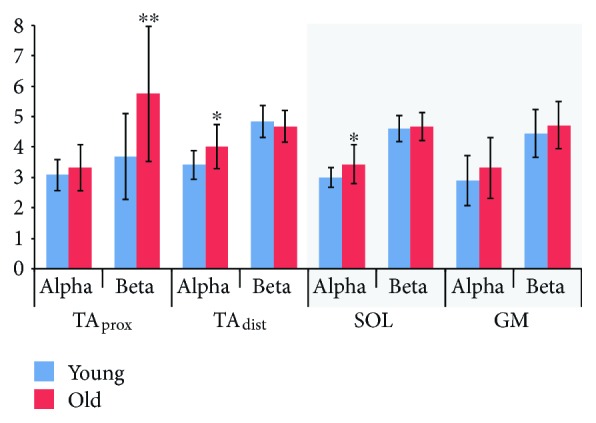
EMG power. Summed EMG power in alpha (5–15 Hz) and beta (15–35 Hz) bands for young and old subjects during dorsiflexion (white background) and plantar flexion (grey background). Significant differences between young and old groups are indicated by ^∗^*p* < 0.05; ^∗∗^*p* < 0.01. TA_prox_: proximal end of anterior tibial; TA_dist_: distal end of anterior tibial; SOL: soleus; GM: medial gastrocnemius.

Figure 4 shows summed EMG power for young and older subjects. Alpha EMG power was greater in old subjects for TA_dist_ (*p* = 0.011) and SOL (*p* = 0.023), whereas group differences were not significant for TA_prox_ (*p* = 0.312) or GM (*p* = 0.218). In the beta band, old subjects had significantly greater power for TA_prox_ (*p* = 0.005), but no other differences were detected (TA_dist_, *p* = 0.413; SOL, *p* = 0.666; GM, *p* = 0.363).

### 3.2. Coherence during Static Dorsiflexion

**Figure 5:**
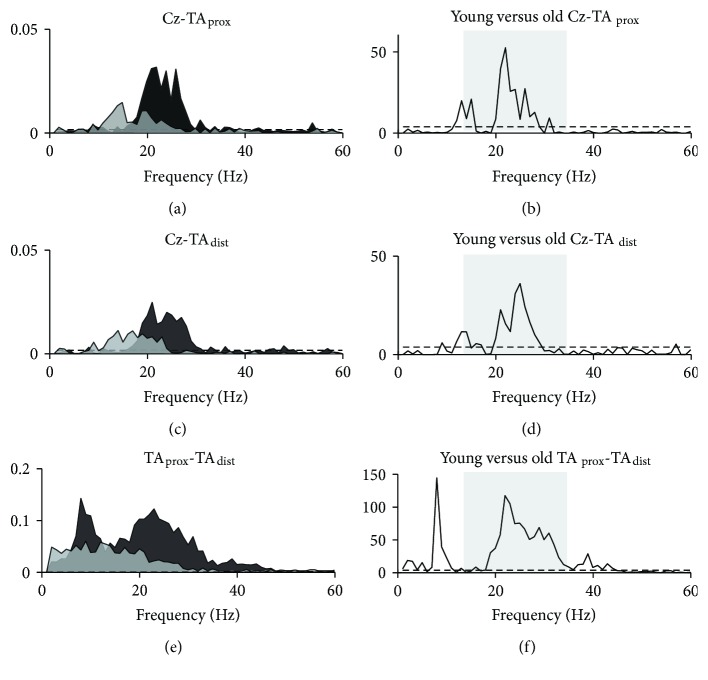
Pooled coherence during dorsiflexion. Pooled coherence estimates for young (black areas) and old (light grey areas) groups during static dorsiflexion (a, c, e) and *χ*^2^ test statistics as a function of frequency (b, d, f) illustrating frequencies at which group means differed. Shaded boxes in b, d, and f mark the beta band (15–35 Hz). Dashed lines indicate upper 95% confidence interval limits. TA_prox_: proximal end of anterior tibial muscle; TA_dist_: distal end of anterior tibial muscle.

Pooled coherence for young and old groups during the dorsiflexion task is presented in Figure 5. For both groups, pooled beta coherence estimates exceeded significance levels for cortico- and intramuscular coherence measures and indicated greater coherence in young than in old subjects (Figures 5(a), 5(c), and 5(e)). The *χ*^2^ extended difference of coherence test confirmed that group means differed significantly at beta frequencies for both cortico- and intramuscular coherence (Figures 5(b), 5(d), and 5(f)). The *χ*^2^ test also revealed significant group differences in intramuscular coherence in the alpha band (~10 Hz), where young subjects had greater coherence than old subjects.

**Figure 6:**
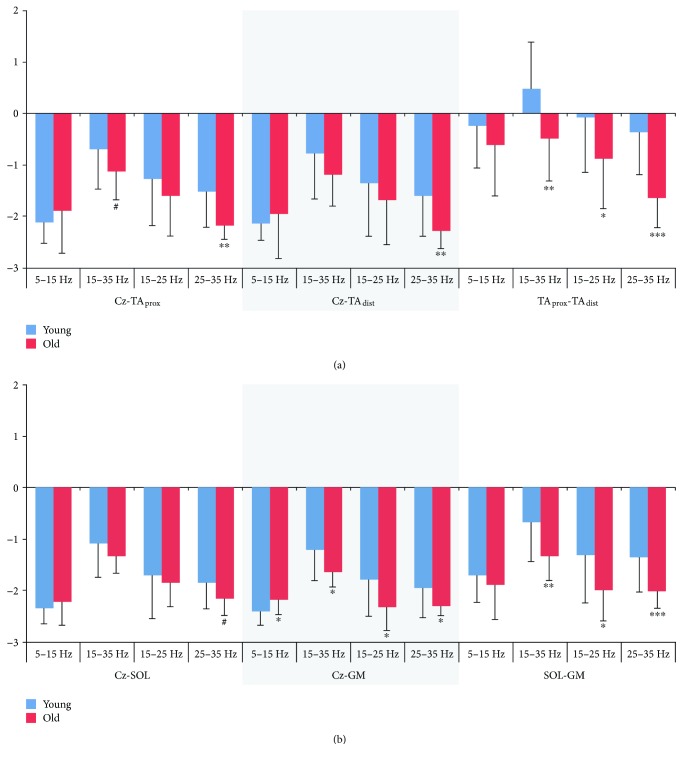
Coherence area estimates. Logarithmic coherence area in alpha (5–15 Hz), beta (15–35 Hz), low (15–25 Hz), and high (25–35 Hz) beta during static dorsiflexion (a) and plantar flexion (b) for young and old subjects. Significant differences between young and old groups are indicated by ^∗^*p* < 0.05; ^∗∗^*p* < 0.01; ^∗∗∗^*p* < 0.001; ^#^*p* < 0.1. TA_prox_: proximal end of anterior tibial; TA_dist_: distal end of anterior tibial; SOL: soleus; GM: medial gastrocnemius.

Estimates for coherence area in alpha, beta, and high and low beta bands are presented in Figure 6. Differences between young and old subjects were most consistent in the high beta band, where coherence area was greater for young than old subjects for all three coherence measures, though group differences in total and low beta area were also significant for TA_prox_-TA_dist_ coherence and tended towards significance for the total beta band in Cz-TA_prox_. Using this approach, no differences between young and old subjects were present in the area of alpha coherence.

Visual inspection of pooled group plots suggested that coherence in old adults appeared to be slightly shifted toward earlier frequencies, but review of single-subject plots indicated that this trend was driven by three older subjects showing pronounced coherence at alpha to early beta frequencies. In addition, the frequency at which peak beta coherence occurred did not differ significantly between groups for corticomuscular coherence (Cz-TA_prox_, *p* = 0.420, Cz-TA_dist_, *p* = 0.742), although it tended to be lower in old subjects for intramuscular coherence (TA_prox_-TA_dist_, *p* = 0.088).

No significant effects of gender (all *p* > 0.2) or footedness (all *p* > 0.3) on beta band coherence area during dorsiflexion were present.

### 3.3. Coherence during Static Plantar Flexion

**Figure 7:**
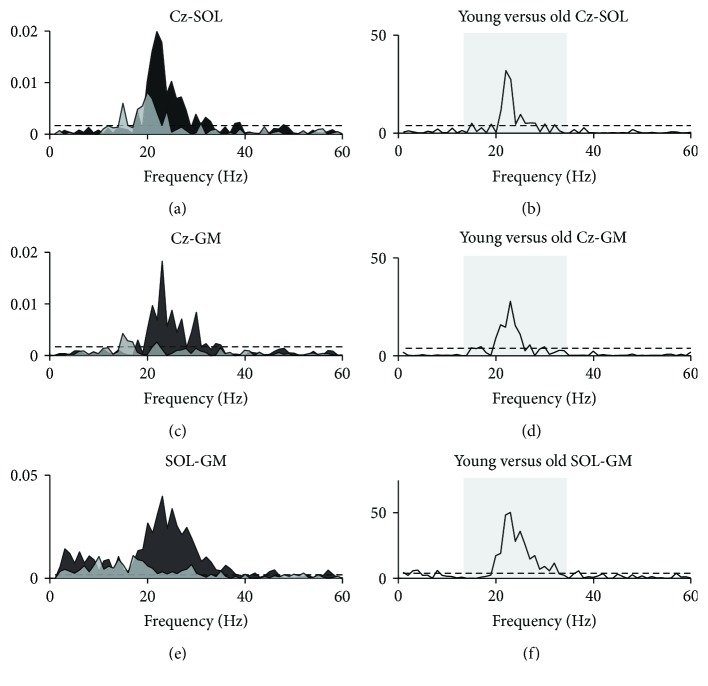
Pooled coherence during plantar flexion. Pooled coherence estimates for young (black areas) and old (light grey areas) groups during static plantar flexion (a, c, e) and *χ*^2^ test statistics as a function of frequency (b, d, f) illustrating frequencies at which group means differed. Shaded boxes in b, d, and f mark the beta band (15–35 Hz). Dashed lines indicate upper 95% confidence interval limits. SOL: soleus; GM: medial gastrocnemius.

Pooled coherence data from the plantar flexion task demonstrated trends similar to those observed for the dorsiflexion task, though amplitudes were lower in both age groups (Figures 7(a), 7(c), and 7(e)). Group estimates indicated statistically significant beta coherence in both young and old subjects for all three measures. Pooled plots suggested that the magnitude of beta coherence was consistently larger for young subjects, which was corroborated by the *χ*^2^ extended difference of coherence test (Figures 7(b), 7(d), and 7(f)). For intermuscular coherence, pooled estimates indicated the presence of alpha coherence in both age groups, but group differences at these frequencies did not reach the significance level. Thus, the *χ*^2^ test indicated that group differences were confined to the beta band.

The area of beta coherence differed between groups in a more widespread manner than for dorsiflexion (Figure 6); Cz-SOL coherence only tended to be greater in young subjects for the high beta band, whereas for Cz-GM and SOL-GM, significant differences in total, low, and high beta bands were observed. In the alpha band, a significant group difference was observed for Cz-GM, where coherence area was greater in old subjects.

Similar to what was observed in dorsiflexion, pooled coherence data suggested that coherence in old subjects was present at slightly lower frequencies than in young subjects. Upon inspection of individual coherence estimates, it was again apparent that the same three old subjects were responsible for this trend. Correspondingly, the frequency at which peak beta coherence occurred did not differ in young and old adults for Cz-GM (*p* = 0.754) or SOL-GM (*p* = 0.673), but a tendency towards lower frequency beta coherence in older adults was present for Cz-SOL (*p* = 0.081).

As for dorsiflexion, beta band coherence during plantar flexion was neither affected by gender (all *p* > 0.4) nor footedness (all *p* > 0.3).

### 3.4. Task Performance and Associations with Power and Coherence

**Figure 8:**
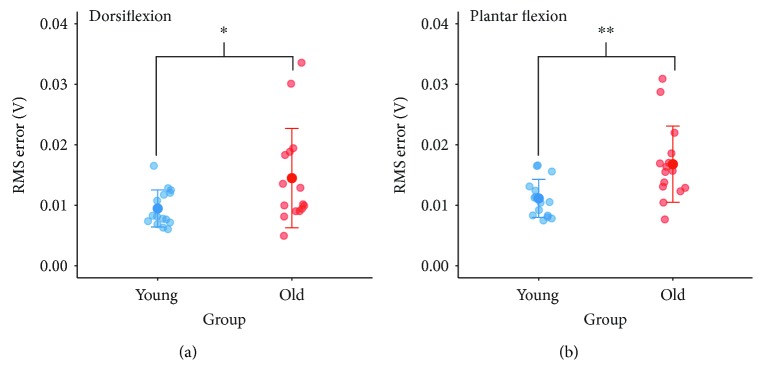
Performance during static contractions. Group differences in force precision during submaximal dorsiflexion (a) and plantar flexion (b). Significant differences between young and old groups are indicated by ^∗^*p* < 0.05, ^∗∗^*p* < 0.01. RMS: root mean square.

Figure 8 shows group differences in force precision during static dorsi- and plantar flexion contractions. RMS error during dorsiflexion was greater for old (14.5 ± 8.2 mV) than for young (9.5 ± 3.1 mV) adults (*p* = 0.035). During plantar flexion, RMS error for old adults (16.8 ± 6.3 mV) was likewise greater than for young adults (11.1 ± 3.1 mV; *p* = 0.004).

To examine if low-frequency rhythmicity in EMG bursts was related to task performance, associations between EMG alpha power and RMS error were explored. EMG alpha power in TA_prox_ was positively associated with RMS error (*r* = 0.47, *p* = 0.008), and a tendency towards a positive correlation between TA_dist_ and RMS error was also present (*r* = 0.36, *p* = 0.054), while alpha power in SOL and GM was not related to RMS error during plantar flexion (SOL, *p* = 0.323, GM, *p* = 0.776).

Furthermore, we investigated possible associations between coherence estimates and performance. Surprisingly, neither cortico- nor intramuscular beta coherence area was correlated with RMS error during the dorsiflexion task (Cz-TA_prox_, *p* = 0.783; Cz-TA_dist_, *p* = 0.745; TA_prox_-TA_dist_, *p* = 0.306), suggesting that the amount of beta coherence was not related to task performance. For the plantar flexion task, RMS error was also unrelated to beta coherence area (Cz-SOL, *p* = 0.327; Cz-GM, *p* = 0.870; SOL-GM, *p* = 0.946). Correlations between RMS error and low and high beta coherence were similarly nonsignificant for both dorsi- and plantar flexion tasks (range for *p* values: 0.175–0.971). Interestingly, alpha coherence area was positively correlated with RMS error for both tasks in five out of six coherence measures (range for *p* values: 0.010–0.062, range for *r*: 0.34–0.46), demonstrating that greater coherence in the alpha band was accompanied by less precision in force production across groups.

## 4. Discussion

The aim of this study was to examine age-related changes in cortico-, intra-, and intermuscular beta band coherence in lower leg muscles during static dorsi- and plantar flexion. We found that beta band coherence estimates were significantly lower in old versus young adults for all coherence measures during both tasks. Error during force tasks was greater for old than young subjects, but no associations were observed between task performance and coherence area in the beta band. Collectively, our results suggest an age-related reduction in oscillatory corticospinal activity in the beta band during a simple task requiring steady state motor output, but the amount of beta coherence was not related to precision in force production. Interestingly, our data indicate a potential effect of alpha coherence and EMG alpha power on task performance.

### 4.1. Beta Band Coherence

For subjects with significant coherence, cortico-, intra-, and intermuscular coherence was most prominent in the beta band, which agrees with previous results demonstrating beta coherence during isometric contractions [10, 11, 13–17, 20]. That this oscillatory coupling in the beta band specifically reflects task-related corticospinal activity is confirmed by multiple lines of evidence. In the monkey, it has been demonstrated that local field potentials in the primary motor cortex are coherent with EMG in the beta band during static contractions, and that these local field potentials were phase-locked with pyramidal cell firing, indicating that corticomuscular coherence is, in the least, partially mediated by the corticospinal tract [39]. Moreover, beta coherence is weakened or absent in patients with central stroke [40]. The use of EEG, MRI, and TMS in combination has also shown, in healthy humans, that corticomuscular coherence is localized at the same site as the muscle's hotspot for TMS [41]. Finally, it has been shown that subthreshold TMS increases corticomuscular coherence, suggesting a role for cortical cells in the generation of beta band coherence [42]. Cortico-, intra-, and intermuscular coherence are thought to generally reflect the same oscillatory corticospinal activity [19], but when considering the indirect measures of intra and intermuscular coherence, contributions from other common neural drives cannot be excluded.

### 4.2. Age-Related Changes in Beta Band Coherence

Our findings of reduced beta band coherence in older subjects are in line with results from a previous study suggesting weakened corticomuscular coherence in older adults during static contractions of arm muscles [23], although other studies have demonstrated unchanged or greater beta coherence in older adults [24–28]. These inconsistencies are not easily reconciled, but considering that beta coherence is task-dependent, differences in task details across studies are likely of utmost importance. One study examined intermuscular coherence in ankle muscles during a simple motor task across the adult lifespan and found no evidence of age-related changes in beta coherence amplitude [26]. However, this study utilized a task consisting of brief phasic dorsiflexions, where visual feedback of EMG traces was provided, but where contraction strength was not controlled, which may contribute to explaining the seemingly divergent results. What is more, this study only examined the corticomuscular coupling indirectly, that is, via intermuscular coherence.

Another recent study investigated intermuscular coherence in triceps surae muscles during stance and found greater beta band coherence in older versus young adults during the uni- but not bipedal posture [27]. These findings indicate that older adults deployed greater oscillatory corticospinal activity during a demanding task, possibly as a strategy to manage their increased postural sway. Interestingly, beta coherence was not greater in older adults during the simpler task, that is, bipedal stance, suggesting that task difficulty may modulate the age-related mobilization of oscillatory corticospinal activity. Our task differed considerably from uni- and bipedal stance, making direct comparisons difficult, but one interesting factor may be differences in the costs of failure in task performance; for static dorsiflexion, there is no consequence to poor performance, whereas during unipedal stance, there is likely a perceived risk of falling that could affect the neural strategy employed.

The age-related reduction we observed in beta coherence, indicative of decreased oscillatory corticospinal activity, is plausibly related to both structural and functional declines in the nervous system. There does not appear to be an age-related loss of neurons in the primary motor cortex [43], but the deterioration in white matter microstructure that occurs with aging likely affects corticospinal connectivity [4, 5]. Decreases in synaptic density [44] and dendritic arborization [45] may likewise contribute to reduced corticomuscular coherence in older adults.

Age-related changes in the functional organization of sensorimotor networks have also been demonstrated. Functional MRI studies suggest that aging entails greater and more widespread cortical activation including regions involved in sensory processing and cognitive control, even during simple motor tasks [46–48]. The reduction we observed in functional corticospinal involvement is not necessarily inconsistent with these findings but may rather be a consequence of age-related de-differentiation of networks, that is, more defocalized and nonspecific activation patterns may entail a less focused and effective corticospinal coupling. Alternatively, decreased corticospinal involvement may represent a fundamental decline in the sensorimotor system compelling compensatory activation in, for example, cognitive or sensory processing regions. Synchronous activity has been theorized as a particularly efficient mode of corticospinal interaction in tasks with relatively low computational demands [39, 49]; accordingly, decreased coherence in older adults may indicate decreased efficiency in the corticospinal system necessitating compensatory strategies in order to perform a given task. The relationship between age-related reductions in beta coherence and modifications of sensorimotor networks should be explored in future studies.

When we examined group estimates for pooled coherence, we noticed that the area of significant coherence was shifted towards lower frequencies (late alpha-early beta) in older subjects. However, this shift appeared to be primarily driven by three older subjects and was not statistically evident when comparing the frequency for peak beta coherence in old versus young groups. We only investigated this apparent shift within the confines of the beta band, as the origin of this activity is well-established, but it is possible that the frequency band reflecting oscillatory corticospinal activity shifts slightly with aging and is thus present at somewhat lower frequencies (~10–25 Hz). This would be in line with previous results indicating that cortical activity and corticomuscular coherence slow with aging [24]; thus, this potential shift towards lower frequencies should be explored in further projects.

It should be noted that we found higher BMI in the older subjects, but we do not believe that this could explain the observed differences in coherence. Although more subcutaneous fat could affect the EMG amplitude, it would not be expected to affect the frequency of the signals, which is what is used for coherence analyses.

### 4.3. Mechanisms Contributing to Decreased Performance in Older Subjects

The functional significance of the observed decline in beta band coherence is unclear. We expected that greater oscillatory corticospinal activity would be positively related to precision in force production, as suggested by a previous study [49], but our results indicated no association between beta coherence and force precision; older adults demonstrated greater error and less beta coherence, but the two parameters were not related. As a more challenging visuomotor task was used by Kristeva and colleagues [49], requiring subjects to respond precisely to an applied force of 4% MVC, this divergence may reflect that our task was not sufficiently demanding to detect a positive effect of beta coherence on performance. On the other hand, other studies have found that greater beta coherence was associated with worsened precision [14, 25], which emphasizes that the functional significance of beta coherence probably also differs according to task demands.

We also investigated the presence of age-related physiological (or essential) tremor as a possible explanation for decreased force precision in older adults [50]. EMG power in the alpha band was greater in TA_dist_ and SOL for old versus young subjects, indicating greater tremor in older subjects in these muscles, as would be expected. Alpha power was also positively associated with RMS error across groups for TA_prox_ and tended strongly to be associated with error for TA_dist_, which suggests that the degree of rhythmic bursting in EMG had a negative effect on the ability to precisely maintain the target force. Interestingly, we also found positive correlations between the area of alpha coherence and RMS error for five out of six coherence measures. It has been suggested that alpha coherence is related to the central component of physiological tremor [22, 51]; however, group differences in the area of alpha coherence were only significant for Cz-GM, indicating that the area of alpha coherence is not a likely explanation for worsened force precision in older subjects but rather a factor contributing to individual differences in the ability to maintain a target force precisely. This is also supported by the observation that young subjects had greater magnitude of intramuscular alpha coherence at ~10 Hz when coherence was compared between groups at each frequency of interest. Altogether, the significance of these correlations is ambiguous, particularly when taking into account the low magnitudes of alpha coherence area estimates, but the consistency of correlations across different muscles and coherence measures warrants further investigation in future studies.

Nonetheless, greater physiological tremor in older adults may by some means be related to the lack of association observed between beta band coherence and force precision. Though speculative, it is plausible that mechanisms leading to increased tremor in older subjects may have a detrimental effect on the ability to effectively regulate force precision via beta oscillations and thus obscure any positive influence of beta coherence on performance.

## 5. Conclusion

We have shown that the amount of beta band cortico-, intra-, and intermuscular coherence in ankle muscles is reduced in old versus young adults during static contraction, suggesting an age-related decrease in the strength of oscillatory corticospinal activity during steady-state motor output. Our data also indicate a potential effect of alpha band coherence and tremor on precision control of muscle force. These results may be instrumental in developing new preventive and therapeutic interventions that may strengthen sensorimotor control in elderly subjects. Future projects should focus on delineating the relationship between beta band coherence and specific motor deficits in older adults and exploring mechanisms underlying the observed reduction in effective corticospinal coupling.

## Figures and Tables

**Table 1:** Subject characteristics.

Group	Young (*n* = 15)	Old (*n* = 15)
Age (years)	22.1 ± 1.7	68.3 ± 2.7
Gender (M/F)	7/8	7/8
Body mass (kg)	72.95 ± 15.89	81.45 ± 14.12
Height (m)	1.76 ± 0.08	1.73 ± 0.11
BMI	23.43 ± 4.29	27.31 ± 4.14^∗^
MMSE (score out of 30)	29.0 ± 1.1	28.9 ± 1.0
Footedness (R/L/B)	11/1/3	10/1/4

Values are presented at mean ± standard deviation where applicable. Significant differences between young and old groups are indicated by ^∗^*p* < 0.05. MMSE: Mini-Mental State Examination. Footedness indicates foot preference. R: right; L: left; B: both.
